# Different Expression of Extracellular Signal-Regulated Kinases (ERK) 1/2 and Phospho-Erk Proteins in MBA-MB-231 and MCF-7 Cells after Chemotherapy with Doxorubicin or Docetaxel

**Published:** 2012

**Authors:** Aliakbar Taherian, Tahereh Mazoochi

**Affiliations:** *1- Kashan Anatomical Research Centre, Kashan University of Medical Science, Kashan, Iran*

**Keywords:** Breast Cancer, Docetaxel, Doxorubicin, MCF-7, MDA-MB-231, Phospho-ERK

## Abstract

**Objective(s):**

Curative treatment of breast cancer patients using chemotherapy often fails as a result of intrinsic or acquired resistance of the tumor to the drug. ERK is one of the main components of the Ras/Raf/MEK/ERK cascade, which mediates signal from cell surface receptors to transcription factors to regulate different gene expression. In this study, cytotoxicity and the expression of Erk1/2 and phospho-ERK was compared in MDA-MB-231 (ER-) and MCF-7 (ER+) cell lines after treatment with doxorubicin (DOX) or docetaxel (DOCT).

**Materials and Methods:**

Cell cytotoxicity of DOX or DOCT was calculated using MTT assay. Immonofluorescent technique was used to show MDR-1 protein in MDA-MB-231 and MCF-7 cells after treatment with DOX or DOCT. The expression of ERK1/2 and phpspho-ERK was assayed with immunoblotting.

**Results:**

Comparing IC_50_ values showed that MDA-MB-231 cells are more sensitive than MCF-7 cells to DOX or DOCT. Immonofluorescent results confirmed the expression of MDR-1 in these two cell lines after DOX or DOCT treatment. In MDA-MB-231 cells the expression of ERK1/2 and phospho-ERK was decreased after DOX treatment in a dose-dependent manner. In contrast in MCF-7 cells the expression of ERK1/2 and phospho-ERK was increased after DOX treatment. DOCT treatment demonstrated the same result with less significant differences than DOX.

**Conclusion:**

The heterogeneity seen in cell lines actually reflects the heterogeneity of breast cancers. That is why, patients categorized in one group respond differently to a single treatment. These results emphasize the importance of a more accurate classification and a more specific treatment of breast cancer subtypes.

## Introduction

Breast cancer is one of the most common malignancies and the main cause of cancer death in women between 40-55 years old (1). Breast cancer is a very heterogenic disease and this feature renders its treatment complexity. Most of the time patients with the same diagnostic and clinical prognostic profile respond very differently to the same treatment (2). Most likely this is because of our poor current classification of breast cancer that is based mainly on morphology (3-5). In cancer treatment, chemotherapy refers to the use of cytotoxic drugs to kill or slow the growth of rapidly multiplying cancerous cells. DOX is a anthracycline-based chemotherapeutic drug widely used for the treatment of metastatic breast cancers (6, 7). The exact mechanism of DOX action is not clear but it is known to interact with DNA double helix and stop the process of replication (8, 9). Another drug is DOCT which is a Texan-class of chemotherapy drug (10). DOCT is an antimitotic drug widely used for the treatment of breast, ovarian, and non-small cell lung cancer (10). DOCT binds to microtubules, stabilizes and prevents their depolymerization (10, 11). Although standard treatment for advanced breast cancer is chemotherapy but often, curative treatment of cancer patients fails as a result of intrinsic or acquired resistance of the tumor to chemotheraputic agents. As a result of cross-resistance, resistance of tumors occurs to a whole range of drugs with different structures (multi-drug resistance) (12). Multi-drug resistance limits the effectiveness of chemotherapy and is responsible for the poor efficacy of breast cancer chemotherapy (13, 14). Thus, an intense research is underway to study the genetic and molecular mechanism of chemotherapy. 

The major protein kinase signaling pathways play important roles in different cell activities like proliferation, differentiation and apoptosis. So the activation or inactivation of a variety of signaling pathways in cancer cells are involved in drug resistance (15, 16). The Ras/Raf/MEK/ERK cascade mediates signal from cell surface receptors to transcription factors to regulate gene expression. Mutation or over expression of upstream molecules such as epidermal growth factor receptor (EGFR) induces the activity of this pathway in some tumors. Also by phosphorylation of apoptotic regulatory molecules like Bad, Bcl-2 and caspase 9, this pathway is involved in the regulation of apoptosis (17). In Raf/MEK/ERK pathway, ERK1/2 is one of the principle signal protein molecules, which have been widely investigated to validate its use as a drug target (18, 19). 

The purpose of this study was to compare the expression of ERK1/2 and phospho-ERK in two different breast cancer cell lines (MDA-MB-231 and MCF-7) after treatment with DOX or DOCT. MDA-MB-231 cells do not express estrogen receptor (ER-) and have a high invasive activity *in vitro* (20-23). MCF-7 cells have low invasive capability (21, 24) and express both, estrogen receptor (ER+) (20, 25), and progesterone receptor (20). This study wishes to provide a comparative molecular characterization of chemotherapy treatment in the breast caner MDA-MB-231 and MCF-7 cell lines. 

## Materials and Methods

Antibodies against ERK1/2 and the HRP secondary antibodies were obtained from Santa Cruz Biotechnology. The phospho-ERK antibody was from cell signaling. DOX and DOCT were purchased from Sigma.

***Breast cancer cell lines***

MDA-MB-231 and MCF-7 cells were purchased from Pasture Institute of Iran. MDA-MB-231 and MCF-7 were cultured in RPMI 1640 containing 10% FBS and 100 Uml penicillin/streptomycin. Cells were grown at 37 C in a humidified incubator with 5% CO2.

***MTT assay***

A colorimetric assay involving the tetrazolium salt was used to assess the antiproliferative effects of DOX or DOCT. Briefly, 96-well plates were seeded with 5000 cell/well of breast cancer cells and allowed to grow for 24 hrs. Cells were treated with different concentrations of DOX or DOCT for 48 hrs. Then 10 ul of MTT (3-(4,5-dimethylthiazol-2-yl)-2,5-diphenyltetrazolium bromide, 5 mg/ml in phosphate buffered saline (PBS)) was added to each well at 37 C for 4 hrs. The foramazon crystals were resolved with DMSO and the absorbance of the wells for 570 nm was measured with a micro plate reader. 

All experiments were carried out more than three times, and the data is expressed as means ± standard deviation. Statistical analysis of the data was performed using Excel program. IC_50_ values (i.e. IC_50_ value is the half maximal inhibitory concentration) were estimated from *in vitro* dose–response curves using a linear regression analysis.

***Immunofluorescence***

Breast cancer cells were cultured on cover slips in cell culture plates. Cells were treated with different concentration of DOX or DOCT (0, 1, 3, 14 μM) and incubated for 48 hrs. Cells were washed with PBS and fixed with 4% Paraformaldehyde/PBS for 15 min at room temperature. Cells were washed and subjected to immunofluorescence staining with primary and secondary antibodies (anti-MDR-1: sc-55510 and goat anti-mouse IgG-FITC: sc-2010). 

***Western blotting***

Cell plates were grown to around 95% confluency, washed once with cold PBS and lysed with 500 µl of RIPA buffer (50 mM Tris, pH 8, 150 mM NaCl, 0.1 % SDS, 0.5 % Na deoxycholic acid, 1% NP-40 or IGEPAL, 10 g aprotinin per ml, and 10 g leupeptin per ml). Cells were scrapped and broken down with a 25 G 5/8 needle. The cell extract was centrifuged for 30 min at 14000 rpm and the supernatant was kept at -20 C. Protein concentration was assayed with Bradford method. Equal amounts of protein (24 gwell) were electrophoretically separated in SDS polyacrylamide gels and proteins were transferred onto nitrocellulose membrane with a semi-dry gel transfer apparatus. Membranes were blocked with 5% milk in PBST (PBS with 0.05% Tween 20) for 1-2 hr at rt. Membranes were incubated with primary antibody at 4 C over night. Then washed for 10 min with PBST and incubated with HRP-labeled antibody in 5% milk in PBST for 1 hr. Membranes were washed for 20-30 min with PBST, treated with chemiluminescence reagents and exposed to Kodak film. 

## Results

***Drug cytotoxicity effect***

MTT assay technique was used to assess the cytotoxicity effect of DOX or DOCT in MDA-MB-231 and MCF-7 cells. Cells cultured in 96-well plates were treated with different concentrations of Dox or DOCT for 48 hrs. IC_50_ value was estimated from dose-response curves obtained following 48 hrs exposure to DOX or DOCT.

Results showed that IC_50_ of Dox in MDA-MB-231 was lower than that in MCF-7 cells (Table 1). The IC_50_ of DOCT was also calculated for both cell lines using MTT assay. Results showed a lower IC_50_ of DOCT in MDA-MB-231 than in MCF-7 as well (Table 1). Generally the IC_50_ of DOCT was lower than DOX in both cells (Table 1).

**Table 1:** IC_50_ of DOX and DOCT in MDA231 and MCF-7 cells. Cells were seeded in 96-well plates. Different concentration of each drug was added to different wells and after 48 hrs live cells were assayed by MTT assay

	**IC**_50_			
	Doxorubicin (nM)		Docetaxel (nM)	
Cells	Mean	STDEV	Mean	STDEV
MDA-MB-231	887.75	65.26	634.58	92.4
MCF-7	1189.47	101.00	762.82	18.47

***MDR-1 expression in breast cancer cell lines after chemotherapy***

In resistant cells to chemotherapy, there is an increased expression of *mdr* genes, which leads to overproduction of MDR protein (26, 27). 

**Figure 1:**
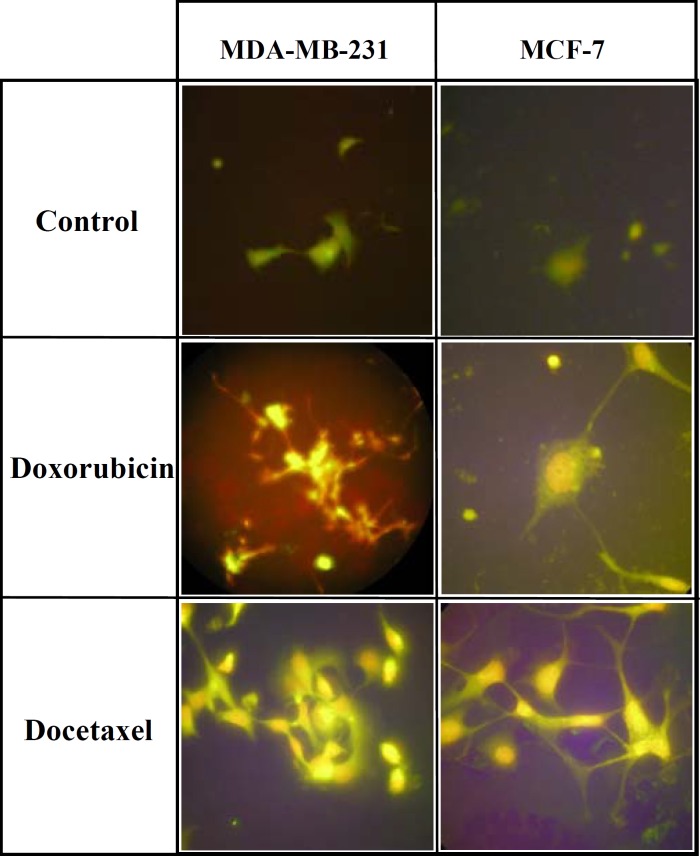
The expression of MDR-1 protein in MDA-MB-231 and MCF-7 cells after DOX or DOCT treatment. Breast cancer cells were cultured on cover slips and after 48 hrs treatment with DOX or DOCT, the expression of MDR protein was assayed by immunoflourescence technique

The aim of this study was to assay the activation of ERK1/2 in breast cancer cells when they are responding to chemotherapy. So immunocytochemistry was used to determine whether the DOX-treated MDA-MB-231 or MCF-7 cells are associated with increased expression of MDR. Parental MDA231 or MCF-7 cells did not express MDR protein significantly. In contrast, MDR protein was highly expressed in both cell lines after drug treatment (Figure 1) 

***ERK and phospho-ERK expression after DOX treatment***

**Figure 2:**
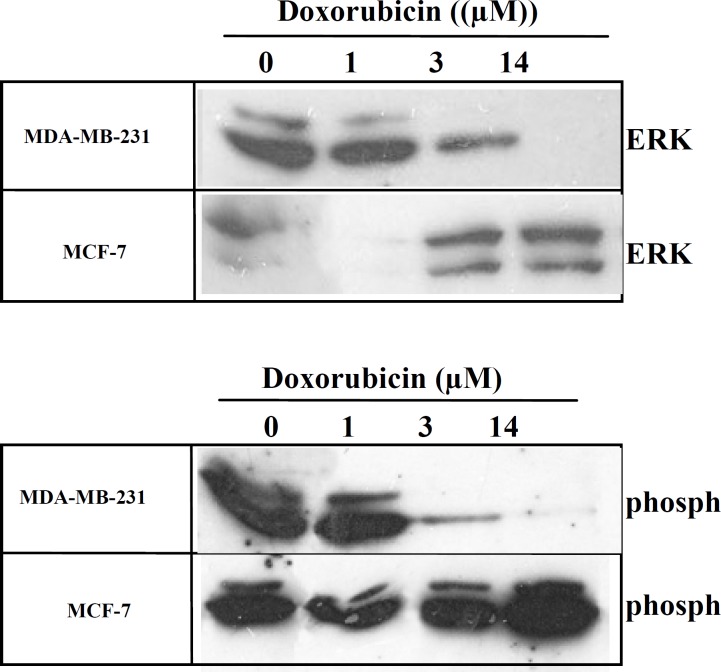
The expression of ERK1/2 and phospho-ERK in MDA-MB-231 and MCF-7 cell lines after doxorubicin treatment. Breast cancer cells in plates were treated with different concentrations of doxorubicin for 48 hr. Total protein was extracted and equal amount of proteins were subjected to immunoblotting with anti-ERK or anti-phospho-ERK antibodies

In this study we investigated the expression of ERK1/2 and phospho-ERK in breast cancer cells after treatment with either DOX or DOCT. Equal number of cells were seeded in 6 cm plates and incubated at 37 C, to grow to a monolayer cells with an approximately 75% confluency. Plates were treated with different concentrations of DOX or DOCT for 48 hrs. Pilot experiments were done to find the right concentration of drugs in order to have enough cells left after 48 hrs for total protein extraction. Equal amounts of different protein samples were separated with poly-acrylamid gel and after transferring proteins to nitrocellulose membrane they were blotted with antibodies against ERK1/2 or phospho-ERK. Figure 2 shows the expression levels of ERK1/2 and phospho-ERK proteins after DOX treatment. Both MDA-MB-231 and MCF-7 cells show ERK1/2 and phospho-ERK expression in control samples that changed dramatically after treatment with DOX. In MDA-MB-231 cells the expression of ERK1/2 and phospho ERK1/2 decreased by increasing drug concentration. In MDA-MB-231 cells the expression of ERK1/2 and phospho-ERK at 1 µM concentration was lower than control sample, and at 3 µM concentration it decreased further until it disappeared at 14 µM of DOX. 

In contrast in MCF-7 cells the lower concentration of DOX caused a decrease in the expression of both ERK1/2 and phospho-ERK but the higher concentration (3 and 14 µM) induced their expression to a higher level. This dose dependent increasing expression was more obvious for phospho-ERK than for ERK.

***ERK and phospho-ERK expression after DOCT treatment***

**Figure 3:**
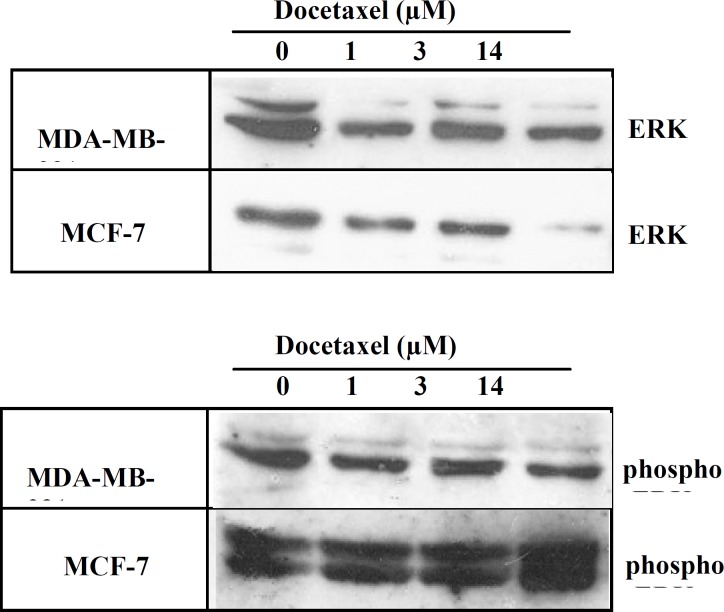
The expression of ERK1/2 and phospho-ERK in MDA-MB-231 and MCF-7 cell lines after docetaxel treatment. Breast cancer cells in plates were treated with different concentrations of docetaxel for 48 hrs. Total protein was extracted and equal amount of proteins were subjected to immunoblotting with anti-ERK or anti-phospho-ERK antibodies.

The expression of ERK1/2 and phospho-ERK was compared in MDA-MB-231 and MCF-7 cells after DOCT treatment as well. Figure 3 shows the expression of ERK1/2 and phospho-ERK in both cell lines after treatment with DOCT. In MDA-MB-231 cells the expression of ERK1/2 decreased by DOCT treatment although increasing concentration of the drug did not cause a steady trend of decreasing expression. For phospho-ERK a decreased expression was seen after DOCT treatment but the difference was less significant than ERK1/2 expression. In MCF-7 cells DOCT drug caused a decreased expression for ERK1/2 and an increased expression for phospho-ERK (Figure 3). This decreased and increased expression of ERK1/2 and phospho-ERK in MCF-7 cells were more obvious in the maximum concentration of drug. Generally speaking, in both cell lines DOX treatment leads to a better dose-dependent response of ERK1/2 and phospho-ERK expression than DOCT.

## Discussion

Breast cancer is one of the most common cancer and the main cause for women death in the world (1, 28). Chemotherapy is an important therapeutic step in breast cancer treatment. DOX and DOCT are common drugs used for chemotherapy but often patients develop resistance to these drugs (6, 7, 29). Resistance to chemotherapy is a common and still unsolved clinical problem. Studying the signaling pathways involved in cells resistance would supply a better understanding of the mechanism of drug-induced resistance. Breast cancer cell lines provide an unlimited source of material for research and are believed to reflect the heterogeneity of the tumor cells. In this experiment, we compared the expression of ERK1/2 and phospho-ERK in two different breast cancer cell lines after treatment with Dox or DOCT. Our results showed that these cell lines responded differently to the same chemotherapy drug in a similar situation. First the IC_50_ of DOX and DOCT were assessed for breast cancer cell lines (MDA-MB-231 and MCF-7) using MTT assay technique. Results showed that MDA-MB-231 cells are more sensitive than MCF-7 cells to DOX since the IC_50_ of MDA-MB-231 (887.75 ± 65.26 nM) was less than that for MCF-7 (1189.47 ± 101.00 nM). In contrast MCF-7 cells were more resistant to DOX since a higher concentration of DOX was needed to kill 50% of the cells. The calculated *P-*value (0.038) confirmed the significance of the difference. To test if this different sensitivity belongs to DOX or not, the IC_50_ of DOCT was assayed as well. Results showed that IC_50_ of DOCT for MDA-MB-231 cells (634.58 ± 92.4 nM) was lower than that for MCF-7 cells (762.82 ± 18.47 nM). With a *P-*value of 0.0001, this difference was statistically significant as well. This shows that MDA-MB-231 cells are more sensitive than MCF-7 cells to DOCT as well. DOX cytotoxicity, using MTT assay in other experiments, shows different values ranging from nM to nM. According to Gariboldi *et al*, IC_50_ of Dox in MDA-MB-231 and MCF-7 cells was 503 and 369 nM respectively (30) while in other studies it was reported 10 µM for both of them (31, 32). The reported IC_50_ of DOCT in MDA-MB-231 and MCF-7 cells by other studies show a vide range as well. The IC_50_ of DOCT in MDA-MB-231 was reported 40 nM (33) or 18.7 µM (34) and in MCF-7 cells 59.6 nM (35) or 17.47 µM (34). A possible reason for these discrepancies could be the differences in protocols like, number of cells seeded per well or drug exposure time. Overall, our results of drug cytotoxicity showed that in a similar situation, MCF-7 cells were more resistance to both DOX and DOCT drugs than what have been confirmed in other studies (36, 37). So some intrinsic factors in MDA-MB-231 and MCF-7 cells should be involved in different sensitivity of these two cell lines.

To study the effect of chemotherapy on Raf/MEK/ERK pathway, the expression of ERK1/2 and phospho-ERK was compared in MCF-7 and MDA-MB-231 cells after DOX treatment. In the present study, we have demonstrated that MDA-MB-231 and MCF-7 cell lines did not express MDR-1 but after exposure to Dox, they express high level of MDR-1 as detected by immunofluorescence staining (Figure 1). Based on this finding, we conclude that after drug treatment, the pathways related to drug resistance are activated in these cell lines. Our results also showed that the concentration of drug used for western blot experiments was enough to induce a higher expression of MDR-1 in breast cancer cells (Figure 1). A 3.5 µM of Dox solution is an equivalent concentration of drug normally given to patients (90 mg dose, administered as 50 mg/25 ml, for a patient with a body mass index of 20 kg/m^2^) (36). Results of western blot analysis showed that in MDA-MB-231 cells the expression of ERK1/2 and phospho-ERK decreased after DOX treatment in a dose-dependent manner (Figure 2). Cell proliferation is the most important characteristic of tumor cells. Cancer cells start with atypical hyperproliferation, progress into invasive and finally into metastatic disease. Most studies have indicated that Ras/Raf/MEK/ERK pathway can promote proliferation and malignant transformation (38). For instance in leukaemia, epithelial and many other tumor cells from human patients, ERK1/2 pathways are hyperactivated (39). In some breast cancers a higher expression of phospho-ERK have been correlated to a shorter disease free survival (40). Here the decreased expression of phospho-ERK after drug treatment in our results is in favor of drug toxicicity and cell apoptosis. That is why Raf/MEK/ERK is an important pathway to target for therapeutic interventions. A few inhibitors of Ras, Raf, and MEK have been developed and some have been in clinical trials, too (17). In contrast, in MCF-7 cells the expression of ERK1/2 and phospho-ERK increased after DOX treatment (Figure 2). Although classical studies indicate that higher expression of phospho-ERK can promote proliferation (38), but in some cancers different aspects of phospho-ERK expression have been determined. In prostate cancer the activation of Raf/MEK/ERK pathway promote differentiation, while in hematopoietic cancer, they induces proliferation. So to inhibit cancer growth in prostate cancer this pathway should be induced but in hematopoietic cancer, it has to be inhibited (17).

Overall the higher expression of phospho-ERK in MCF-7 cells after Dox treatment, apparently, opposes the pathway or mechanism of apoptosis. It might be the reason why MCF-7 cells in our experiments were more resistance to DOX than to MDA-MB-231 cells. 

The expression of ERK1/2 and phospho-ERK was compared in MDA-MB-231 and MCF-7 cells after DOCT treatment as well. These two cell lines responded differently to DOCT treatment.

Although the difference was not as significant as was for DOC but it was interesting to see that DOCT treatment induced different responses in these two cell lines as well. The expression of ERK1/2 was decreased in both cell lines after DOCT treatment. The expression of phospho-ERK decreased in MDA-MB-231 but increased in MCF-7 cells. The increased expression of phospho-ERK in MCF-7 after DOCT treatment was not as so significant as it was in DOX treatment (Figures 2, 3). 

DOCT is a well established anti-mitotic chemotherapy drug used for treatment of several types of cancers including breast cancer (10). DOCT binds to microtubules and by stabilizing microtubule assembly and preventing physiological disassembly, inhibits mitosis (10). Beside inhibiting mitosis, DOCT induces phosphorylation of Bcl-2, which lead to apoptosis of cancer cells (10). DOX interact with DNA double helix that results in the inhibition of the process of replication (8, 9). Although the action mechanism of DOCT and DOX is not completely understood, but there are some differences between their action mechanism and these potential differences are the reason that DOCT can be used for treatment of patients who have an advanced or metastatic cancer and for whom anthracycline-based chemotherapy failed to stop cancer progression or relapsed (41). In our study DOCT cytotoxicity was higher than DOX for both cell lines (Table 1). 

In our study, the increased dose-dependent expression of phospho-ERK in MCF-7 cells after DOX treatment was not seen after DOCT treatment (Figure 2 and 3). So the possible intervention of the high expression of phospho-ERK in MCF-7 cells after DOX treatment with apoptosis pathway has been weekend in DOCT treatment. One of the reasons that DOCT works better than DOX in tumor treatment is that it doesn’t induce the expression of ERK and phospho-ERK in cells like MCF-7 cells as DOX does.

Another possibility that chemotherapy in these two cell lines lead to such conflicting results in the activation of ERK may be due to the expression of lineage-specific factors. MCF-7 cell express estrogen receptor (ER) and progesterone receptor (PR) while MDA-MB-231 cells do not (20-23, 25).The expression of ER could be related to the different responses of the MCF-7 cells since several studies verify this hypothesis. According to different studies, roughly 65% of premenopausal and 80% of postmenopausal breast cancers are ER-positive. This suggest that around 75% of breast cancer cases are ER+ (42). Tamoxifen is the most widely used antiestrogen in ER+ breast cancer patients (43). Immunohistochemical analysis on ER+ breast cancer samples after treatment with tamoxifen showed that there is a correlation between activated ERK and ER expression. A positive staining for activated ERK in these patients indicated a better relapse-free survival in women treated with tamoxifen (44). In other experiments tamoxifen resistant cells showed a higher expression of phospho-ERK (43, 45). This is similar to the result that we saw after treatment of MCF-7 with DOX or DOCT (Figures 2, 3). In the ER positive-tumor that are resistant to tamoxifen, there is a high expression of EGFR, and downstream proteins such as ERK1/2 (46-48). So the higher expression of phospho-ERK after chemotherapy is rather a general characteristic of MCF-7 cells than a drug-specific or experimental-specific result (42). 

## Conclusion

By and large, our experiment showed that different breast cancer cell lines responded differently to a single drug. These results demonstrate the diversity of cellular responses to DOX or DOCT besides expressing MDR-1 protein. The heterogeneity seen in cell lines actually reflects the heterogeneity of breast cancers, that is why, patients categorized in one group respond differently to a similar treatment (49). So different type of breast cancers need different types of treatment including chemotherapy. This result emphasizes the importance of multifactorial analyses of cellular response to chemotherapy. 
